# Exploring the mechanism of agarwood moxa smoke in treating sleep disorders based on GC–MS and network pharmacology

**DOI:** 10.3389/fmed.2024.1400334

**Published:** 2024-05-09

**Authors:** Nianhong Chen, Yucheng Xia, Weiyan Wu, Siyu Chen, Mingming Zhao, Yanting Song, Yangyang Liu

**Affiliations:** ^1^Key Laboratory of Tropical Biological Resources of Ministry of Education, School of Pharmaceutical Sciences, Hainan University, Haikou, China; ^2^Hainan Provincial Key Laboratory of Resources Conservation and Development of Southern Medicine, Key Laboratory of State Administration of Traditional Chinese Medicine for Agarwood Sustainable Utilization, International Joint Research Center for Quality of Traditional Chinese Medicine, Haikou, China; ^3^College of Traditional Chinese Medicine, Hainan Medical University, Haikou, China; ^4^Chengmai County Hospital of Traditional Chinese Medicine, Haikou, China

**Keywords:** agarwood moxa smoke (AMS), agarwood moxa stick, agarwood moxibustion therapy, sleep disorders, GC–MS, network pharmacology, molecular docking

## Abstract

**Background:**

Agarwood moxibustion is a folk therapy developed by individuals of the Li nationality in China. There is evidence that agarwood moxa smoke (AMS) generated during agarwood moxibustion therapy can treat sleep disorders via traditional Chinese medicines’ multiple target and pathway characteristics. However, the specific components and mechanisms involved have yet to be explored.

**Objective:**

GC–MS (Gas Chromatography–Mass Spectrometry) and network pharmacology were used to investigate AMS’s molecular basis and mechanism in treating sleep deprivation.

**Method:**

GC–MS was used to determine the chemical composition of AMS; component target information was collected from TCMSP (Traditional Chinese Medicine Systems Pharmacology), PubChem (Public Chemical Database), GeneCards (Human Gene Database), and DisGeNet (Database of Genes and Diseases) were used to identify disease targets, and JVenn (Joint Venn) was used to identify the common targets of AMS and sleep disorders. STRING was used to construct a protein interaction network, Cytoscape 3.9.1 was used to build a multilevel network diagram of the “core components-efficacy targets-action pathways,” the targets were imported into Metascape and DAVID for GO (Gene Ontology) and KEGG (Kyoto Encyclopedia of Genes and Genomes) analyses and Autodock was used for molecular docking. This research used a network pharmacology methodology to investigate the therapeutic potential of Agarwood Moxa Smoke (AMS) in treating sleep problems. Examining the target genes and chemical constituents of AMS offers insights into the molecular processes and targets of the disease.

**Result:**

Nine active ingredients comprising anti-inflammatory substances and antioxidants, such as caryophyllene and p-cymene, found seven sleep-regulating signaling pathways and eight targets linked to sleep disorders. GC–MS was used to identify the 94 active ingredients in AMS, and the active ingredients had strong binding with the key targets. Key findings included active components with known medicinal properties, such as p-cymene, eucalyptol, and caryophyllene. An investigation of network pharmacology revealed seven signaling pathways for sleep regulation and eight targets linked to sleep disorders, shedding light on AMS’s effectiveness in enhancing sleep quality.

**Conclusion:**

AMS may alleviate sleep disorders by modulating cellular and synaptic signaling, controlling hormone and neurotransmitter pathways, etc. Understanding AMS’s material basis and mechanism of action provides a foundation for future research on treating sleep disorders with AMS. According to the study, Agarwood Moxa Smoke (AMS) may improve sleep quality by modifying cellular and synaptic signaling pathways for those who suffer from sleep problems. This might lead to the development of innovative therapies with fewer side effects.

## 1 Introduction

Sleep disorder syndrome, also known as sleep deprivation, occurs when there is a disruption in the onset and maintenance of sleep, leading to inadequate sleep quality that does not meet the patient’s physiological needs and seriously impacts their daytime activities. This syndrome refers explicitly to circumstances in which it is difficult or impossible to fall asleep, usually when the amount of time spent asleep is inadequate, when it is easy to wake up from sleep, when it is challenging to fall back asleep after waking up, or even when one is awake the entire night (1). Epidemiological surveys indicate that sleep disorders are positively correlated with age and affect approximately 30% of the global population annually. Sleep disorders have become a widespread social problem (2), and the incidence rate of sleep problems in Chinese adults is 42.5%, with a peak incidence rate of 38.2% (3). Sleep disorders not only decrease the quality of life but also the use of benzodiazepine-type Western medicines for treatment, which, although effective quickly, increase the risk of mental and physical diseases, as well as drug dependence and abuse, is dangerous (4, 5). Therefore, there is an urgent need for more effective treatment methods with fewer side effects.

Chinese individuals frequently treat sleep issues with traditional practices such as moxibustion and massage. Traditional Chinese medicines adhere to dialectical treatment and holistic concepts to provide symptomatic treatment for patients with sleep disorders (6). Traditional Chinese medicine’s external therapy approach has fewer adverse effects and is better tolerated by patients (7). This kind of moxa stick is referred to as agarwood moxa stick in this article, and the use of agarwood moxa sticks in moxibustion treatment is known as agarwood moxibustion therapy. Agarwood moxibustion therapy is a traditional Chinese medicine originating from individuals of the Li nationality. This therapy is frequently used to treat a variety of chronic, crippling illnesses, as well as diseases brought on by dampness, wind, and cold; furthermore, this treatment can significantly improve the quality of sleep. Agarwood has long been used as incense in China due to its calming and sleep-promoting properties (8). In traditional Chinese medicine, sleep disorders are thought to be caused by an imbalance among the kidneys, liver, spleen, and heart. The practical volatile components produced by the combustion of agarwood and moxa velvet can be absorbed through the skin, protect the spleen and kidneys, relieve pain, calm the mind, and promote immunity, thereby reducing insomnia symptoms (9, 10). Researchers have examined the components and pharmacological effects of moxa smoke (11, 12), and their findings show that it can lower the levels of aspartic acid and glutamate in the brain.

After burning the agarwood and moxa velvet, the volatile components also have a pleasant, calming effect that can help extend the time spent asleep (13). Sleep problems have resulted from the disruption of healthcare systems, everyday routines, and sleep habits caused by the COVID-19 pandemic. Sleep disturbances are made worse by elements like elevated stress, anxiety, schedule adjustments, and social isolation. Sleep has been further disrupted by uncertainty, fear of infection, and health worries. Screen use has grown during lockdowns and quarantine periods, which has a detrimental effect on sleep quality. Controlling the virus’s transmission and reducing its downstream impacts on public health, such as sleep problems, depend heavily on early forecasting and detection. Addressing these issues and controlling the transmission of the virus are crucial for mitigating the downstream impacts on public health, including sleep problems. Early intervention measures play a pivotal role in this effort, as they enable timely treatment to address both the direct and indirect effects of the pandemic on sleep and overall well-being (14). In some regions of China, agarwood has been used for the prevention of COVID-19, partly based on its reported aromatic purifying properties (15). Therefore, using agarwood moxibustion can not only to some extent achieve early prevention of diseases, but also provide early intervention for sleep problems caused by the spread of COVID-19 (16). While the direct effects of agarwood moxibustion in combating COVID-19 require further scientific validation, its traditional use underscores its potential benefits in public health crises. However, more research needs to be done investigating the components of moxa smoke that are produced during moxibustion treatment and determining how these components affect insomnia. Therefore, to explore the connection between the chemical components of AMS and sleep and to further elucidate the mechanism of AMS-mediated treatment of sleep disorders, in this study, we investigated the practical components of AMS using GC–MS in conjunction with network pharmacology.

## 2 Materials

### 2.1 Medicinal material

The agarwood moxa sticks used in this study were made in the laboratory, with agarwood purchased from Haikou, Hainan, and moxa velvet from Qichun, Hubei. The agarwood slices were crushed, run through a sieve, and mixed evenly with moxa velvet in specific proportions. Then, the samples were laid flat on smokeless paper, rolled tightly, fixed, and finally, a 1.8 cm diameter agarwood moxa stick was made.

### 2.2 Instrument

A Manual SPME Holder (Lot: 155193, Merck, USA), an Agilent gas chromatography-mass spectrometer (Model: 5977B/8860, Agilent Technologies, USA), and a PDMS SPME solid-phase microextraction head (Lot: 163109, Merck, USA) were utilized.

### 2.3 Databases and software

The Traditional Chinese Medicine Systems Pharmacology database and analysis platform (TCMSP)^1^ (17); the PubMed database ^2^(18); the Swiss target prediction database ^3^(19); the Pharmacochemical Database (ChEMBL)^4^ (20); the Universal Protein database (UniProt)^5^ (21); the GeneCards database^6^ (22); the DisGeNet database^7^ (23); the Database for Annotation, Visualization and Integrated Discovery v6.8 (DAVID)^8^ (24); the Jvenn website^9^ (25); the Search Tool for the Retrieval of Interacting Genes/Proteins (STRING)^10^ (26); the Metascape database^11^; and the RCSB Protein Data Bank database (RCSB PDB)^12^ (27) were utilized. Cytoscapev3.9.1 software, Autodock book software, and Pymol software were also utilized.

In summary, the application of Agarwood Moxa Smoke (AMS) in treating sleep disorders is fraught with difficulties, such as problems with data completeness and quality, access to extensive databases, and intricate integration of disparate information. New methods may resist traditional medical practices, and integrating research results into clinical practice may encounter obstacles from regulatory bodies, medical professionals, and patients. Researchers, healthcare professionals, regulatory agencies, and traditional medicine practitioners must work together to remove these obstacles and encourage broader adoption of AMS-based therapy.

## 3 Methods

### 3.1 Collection of AMS

The agarwood moxa stick was ignited and precipitated in a homemade glass collection tank; after 3 min, when the flue gas was complete, a manual sampler was inserted, and the handle was pressed to extend the extraction head. After 10 min of extraction, the extraction head was retracted, and the manual holder was removed. The manual holder was immediately inserted into the gas chromatograph sample inlet (temperature 230°C) for 3 min of analysis without splitting the sample.

### 3.2 GC–MS analysis

An HP-5MS elastic quartz capillary column (30 mm*0.25 mm, 0.25 μm) was utilized. The carrier gas was high-purity helium, with a volume flow rate of 1 mL/min, a sample inlet temperature of 250°C, and a detector temperature of 300°C. Programmed heating was performed as follows: after maintaining a column temperature of 50°C for 1 min, the temperature was raised at a rate of 15°C/min to 143°C, kept for 10 min, raised at a rate of 1°C/min to 155°C, raised at a rate of 25°C/min to 225°C, maintained for 7 min, raised at a rate of 2°C/min to 250°C, and finally maintained for 10 min. The electron bombardment (EI) energy was 70 eV, the ion source temperature was 250°C, the solvent delay was 5 min, and the scanning range was 50–500 amu.

### 3.3 Prediction of the sources and targets of the chemical components in AMS

The traditional Chinese medicine system pharmacology analysis platform (TCMSP) (17) was utilized to search and identify the chemical components in AMS based on the chemical composition data derived from the GC–MS analysis. Traditional Chinese medicine (TCM) uses Agarwood Moxa Smoke (AMS), combining conventional knowledge and cutting-edge scientific techniques. It provides focused treatment for sleep disturbances, which may result in fewer adverse effects and more successful results. By bridging the gap between conventional knowledge and contemporary understanding, scientific validation via GC–MS analysis and network pharmacology increases acceptability and trustworthiness. Because agarwood moxa stick is a traditional Chinese medicine mixture, its smoke contains numerous complex chemical components of traditional Chinese medicine. Based on Lipinski’s Rules of Five, the potential effective active ingredients were identified. For chemical components not found in the TCMSP database, the PubChem database (18) was used to search the Smiles number of the element. Next, an AMS chemical component library was created by searching the pertinent target databases Swiss Target Prediction (19) and the ChEMBL database (20). Finally, the UniProt database (21) was used to standardize target gene names, and only human (Homosapiens) target genes were retained for subsequent analysis.

### 3.4 Identification of targets in sleep disorders

Due to the mind-tranquilizing effects of both moxa velvet and agarwood in agarwood moxa sticks, this study further utilized the Gene Cards (22) and DisGeNet databases (23) to search for genes associated with sleep disorders; “Sleep disorders” was used as the keyword to search for targets in sleep disorders, and gene names were standardized by the DAVID v6.8 database (24). Only pertinent values ≥1.5 were chosen as the primary targets for sleep disorders to ensure the validity of the data.

### 3.5 Construction of the protein–protein interaction network

On the Jvenn platform (25), the targets of AMS components were compared to the disease targets related to sleep disorders, and the shared targets were identified. The shared targets were considered potential targets of AMS for treating sleep disorders. The shared target gene set was input into the STRING database (26). Then, a protein–protein interaction (PPI) network was constructed with *Homo sapiens* as the target species and an average confidence level of 0.4 as the threshold for the interaction score. The PPI network was visualized with Cytoscape v3.9.1 software.

### 3.6 Construction of the “core components-targets-action pathways” network

Cytoscape 3.9.1 software created a multilevel network of “core components, targets, and action pathways” to link AMS’s disease-related genes, core components, and targets. A network was established with circles representing disease-related genes, diamonds representing core components, and triangles representing AMS targets; this network was used to evaluate the mechanism of action of AMS in the treatment of sleep disorders.

### 3.7 GO functional analysis and KEGG pathway enrichment analysis

The targets for treating sleep disorders corresponding to the chemical components of AMS were input into the Metascape and David databases (24). The databases conducted Gene Ontology (GO) and Kyoto Encyclopedia of Genes and Genomes (KEGG) pathway enrichment analyses to obtain biological information about potential targets and analyze AMS’s potential mechanism of action in treating sleep disorders. GO analysis results are divided into the categories of biological processes (BPs), cellular components (CCs), and molecular functions (MFs). According to the *p* values, the top 8 results from GO analysis were selected, and a histogram of enrichment quantity statistics was drawn; for KEGG analysis, the top 7 pathways were selected for visualization.

### 3.8 Molecular docking validation

The active ingredients related to treating sleep disorders by AMS were docked to core targets to forecast and evaluate the protein-molecule interactions and binding energy. After using the TCMSP and PubChem databases to obtain mol2 files of compound structures, the RCSB PDB database (27) was used to obtain PDB files of the core target structures. Using Autodock software (28) for docking and PyMOL software (29) for visualization and processing, binding energy was used as an indicator to evaluate the binding activity and docking effects of ligand-protein interactions. Generally, -1.2 kcal·mol^-1^ binding energy indicates strong binding between the protein and ligand.

## 4 Results

When optimizing computer models to investigate how Agarwood Moxa Smoke (AMS) affects sleep problems, parameter tuning is an essential component. Researchers may increase their models’ efficiency, accuracy, and resilience by fine-tuning their parameters, providing more trustworthy outcomes. Molecular Docking parameter tweaking, route analysis parameters, threshold selection, network visualization, docking method settings, and binding site flexibility are essential tactics. Network quality and dependability are affected by threshold selection, while interpretability is improved by network visualization. Performance assessment, hyperparameter optimization, cross-validation, and stringent validation techniques are all part of overall model optimization.

### 4.1 Chemical composition of AMS

**Figure 1:**
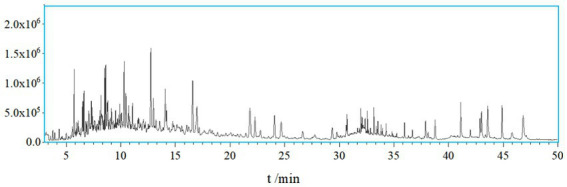
The total ion chromatogram of AMS.

**Figure 2:**
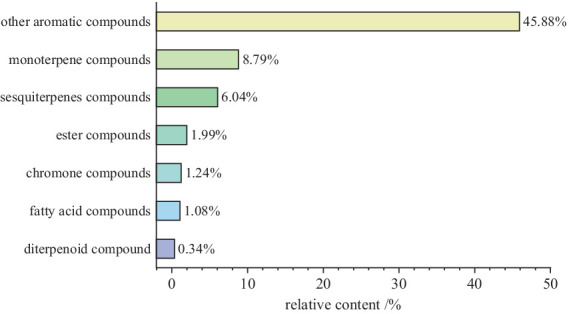
The proportion of various compounds in AMS.

A total of 138 chemical components were identified in AMS. Among these volatile components, there were 20 compounds with a relative abundance of more than 1%, including phenol, 3-methyl-phenol, p-cymene, azulene, endo-borneol, α-terpineol, 4-ethyl-2-methoxy-phenol, caryophyllene, caryophyllene oxide, bis (2-ethylhexyl) phthalate, etc. The components of the smoke and the combustion products of agarwood moxa sticks are complex, and volatile aromatic compounds are one of the main components of AMS. The study looks at how Agarwood Moxa Smoke (AMS) affects sleep problems; however, it has trouble analyzing proprietary or unbalanced datasets. The dependability of findings can be affected by imbalanced datasets, resulting in biased model performance and decreased predicted accuracy. Class distributions can be balanced using ensemble techniques, undersampling, and oversampling. Sensitive information in proprietary datasets makes data access, exchange, and validation difficult. Real-world data assessment requires collaboration with data owners. Scalability testing, result evaluation, and robustness assessment are examples of testing capabilities. Eugenol, 4-phenyl-3-buten-2-one, 4-phenyl-2-butanone, and n-hexadecanoic acid may originate from (30). Sesquiterpene compounds may degrade into monoterpenes and small-molecule volatile compounds after high-temperature cracking. The identified chemicals were mainly phenolic compounds containing methoxy groups and monoterpenoids; these chemicals are related to the aroma produced after the combustion of agarwood moxa sticks (Figures 1, 2; Table 1).

**Table 1:** Effective active ingredients of AMS.

No.	PubChemCID	Compound name	Relative content/%	Chemical formula	Molecular weight/g·mol^−1^
1	1140	Toluene	0.35	C_7_H_8_	92.14
2	7361	2-Furanmethanol	0.17	C_5_H_6_O_2_	98.10
3	7975	2-methyl-Pyridine	0.27	C_6_H_7_N	93.13
4	7929	1,3-dimethyl-Benzene	0.18	C_8_H_10_	106.16
5	7501	Styrene	0.31	C_8_H_8_	104.15
6	14266	2-methyl-2-Cyclopenten-1-one	0.07	C_6_H_8_O	96.13
7	14505	1-(2-furanyl)-Ethanone	0.09	C_6_H_6_O_2_	110.11
8	11565	3,5-dimethyl-Pyridine	0.21	C_7_H_9_N	107.15
9	7668	propyl-Benzene	0.06	C_9_H_12_	120.19
10	240	Benzaldehyde	0.47	C_7_H_6_O	106.12
11	996	Phenol	1.39	C_6_H_6_O	94.11
12	13381	1-Decene	0.25	C_10_H_20_	140.27
13	252324	(Z)-1-Phenylpropene	0.32	C_9_H_10_	118.18
14	9223	Benzofuran	0.29	C_8_H_6_O	118.13
15	7936	2,4-dimethyl-Pyridine	0.30	C_7_H_9_N	107.15
16	14,287	2-ethyl-6-methyl-Pyridine	0.16	C_8_H_11_N	121.18
17	7,463	p-Cymene	1.09	C_10_H_14_	134.22
18	440,917	D-Limonene	0.49	C_10_H_16_	136.23
19	2,758	Eucalyptol	0.81	C_10_H_18_O	154.25
20	335	2-methyl-Phenol	0.51	C_7_H_8_O	108.14
21	342	3-methyl-Phenol	1.03	C_7_H_8_O	108.14
22	991698552	Succinic acid, tridec-2-yn-1-yl 3-methylpentyl ester	0.33	C_23_H_40_O_4_	380.60
23	62385	1-methyl-4-(1-methylethenyl)-Benzene	0.53	C_10_H_12_	132.20
24	14257	Undecane	0.41	C_11_H_24_	156.31
25	6616	Camphene	0.19	C_10_H_16_	136.23
26	8500	1-(4-methylphenyl)-Ethanone	0.22	C_9_H_10_O	134.17
27	8794	Benzyl nitrile	0.43	C_8_H_7_N	117.15
28	159055	(+)-2-Bornanone	0.85	C_10_H_16_O	152.23
29	9231	Azulene	1.61	C_10_H_8_	128.169
30	6552009	endo-Borneol	1.24	C_10_H_18_O	154.25
31	9268	Cyclododecane	1.97	C_12_H_24_	168.32
32	17100	alpha-Terpineol	1.11	C_10_H_18_O	154.25
33	28453	2,6-dimethyl-Undecane	0.31	C_13_H_28_	184.36
34	637759	4-phenyl-3-Buten-2-one	0.44	C_10_H_10_O	146.19
35	29025	Verbenone	0.50	C_10_H_14_O	150.22
36	5372813	2-methyl-3-phenyl-2-Propenal	0.95	C_10_H_10_O	146.19
37	12581	Benzenepropanenitrile	0.50	C_9_H_9_N	131.17
38	17355	4-phenyl-2-Butanone	0.72	C_10_H_12_O	148.20
39	1550846	(E)-3-phenyl-2-Propenenitrile	0.40	C_9_H_7_N	129.16
40	14109	hexyl-Benzene	0.88	C_24_H_38_O_4_	390.60
41	62465	4-ethyl-2-methoxy-Phenol	1.13	C_9_H_12_O_2_	152.19
42	17095	1-Tridecene	2.55	C_13_H_26_	182.35
43	12388	Tridecane	2.11	C_13_H_28_	184.36
44	7055	2-methyl-Naphthalene	0.93	C_11_H_10_	142.20
45	332	2-Methoxy-4-vinyl phenol	0.83	C_9_H_10_O_2_	150.17
46	8817	5-ethenyl-2-methyl-Pyridine	0.54	C_8_H_9_N	119.16
47	7041	2,6-dimethoxy-Phenol	0.62	C_8_H_10_O_3_	154.16
48	5364455	Nonene	0.76	C_9_H_18_	126.24
49	3314	Eugenol	0.46	C_10_H_12_O_2_	164.20
50	14115	heptyl-Benzene	0.61	C_13_H_20_	176.30
51	519194	1-Methyl-4-n-hexylbenzene	0.38	C_13_H_20_	176.30
52	19773	2,6,10-trimethyl-Dodecane	0.56	C_15_H_32_	212.41
53	5352912	1-Tetradecene	3.62	C_14_H_28_	196.37
54	12389	Tetradecane	1.58	C_14_H_30_	198.39
55	11306	1,5-dimethyl-Naphthalene	1.27	C_12_H_12_	156.22
56	11396	2,7-dimethyl-Naphthalene	1.11	C_12_H_12_	156.22
57	5354499	Caryophyllene	2.28	C_15_H_24_	204.35
58	11387	2,6-dimethyl-Naphthalene	1.29	C_12_H_12_	156.22
59	11386	2,3-dimethyl-Naphthalene	0.72	C_14_H_12_O_4_	244.24
60	19774	2,6,10-Trimethyltridecane	1.07	C_16_H_34_	226.44
61	6429347	1,4-Dimethylazulene	0.55	C_12_H_12_	156.22
62	16607	octyl-Benzene	0.55	C_14_H_22_	190.32
63	563197	Cycloisolongifolene	0.89	C_15_H_24_	204.35
64	5364464	Z,Z-3,13-Octadecedien-1-ol	0.60	C_18_H_34_O	266.50
65	25913	1-Pentadecene	2.73	C_15_H_30_	210.40
66	12391	Pentadecane	1.99	C_15_H_32_	212.41
67	6432455	α-Selinene	0.51	C_15_H_24_	204.35
68	13237	2,3,6-trimethyl-Naphthalene	0.98	C_13_H_14_	170.25
69	16479	1,4,6-trimethyl-Naphthalene	0.14	C_13_H_18_	174.28
70	6432640	1H-Cycloprop[e]azulen-7-ol,decahydro-1,1,7-trimethyl-4-methylene-,[1ar-(1a.alpha.,4a.alpha.,7.beta.,7a.beta.,7b.alpha.)]-	0.23	C_15_H_24_O	220.35
71	1742210	Caryophyllene oxide	1.79	C_15_H_24_O	220.35
72	11006	Hexadecane	0.40	C_16_H_34_	226.44
73	11877394	Neointermedeol	0.47	C_15_H_26_O	222.37
74	23217	1-Heptadecene	0.63	C_17_H_34_	238.50
75	12398	Heptadecane	0.29	C_17_H_36_	240.50
76	10719	Chamazulene	0.39	C_14_H_16_	184.28
77	5362709	(Z)-3-Tetradecene	0.59	C_14_H_28_	196.37
78	10,390	Diphenylacetylene	0.31	C_14_H_10_	178.23
79	95724	3-Phenanthrol	0.29	C_14_H_10_O	194.23
80	8217	1-Octadecene	0.44	C_18_H_36_	252.48
81	11635	Octadecane	0.48	C_18_H_38_	252.48
82	79362	Phthalic acid, monooctyl ester	0.35	C_16_H_22_O_4_	278.34
83	6423452	Phthalic acid, butyl tetradecyl ester	0.20	C_26_H_42_O_4_	418.60
84	6782	1,2-Benzenedicarboxylic acid, bis(2-methylpropyl) ester	0.24	C_16_H_22_O_4_	278.34
85	10446	Neophytadiene	0.34	C_20_H_38_	278.50
86	6781	Diethyl Phthalate	0.10	C_12_H_14_O_4_	222.24
87	16221	Dimethyl palmitamine	0.77	C_18_H_39_N	269.50
88	29075	1-Nonadecene	0.19	C_19_H_38_	266.50
89	8222	Eicosane	0.11	C_20_H_42_	282.50
90	985	n-Hexadecanoic acid	0.40	C_16_H_32_O_2_	256.42
91	8907	Isopropyl palmitate	0.08	C_19_H_38_O_2_	298.50
92	3015374	Henicos-1-ene	0.08	C_21_H_42_	294.60
93	7641	Hexanedioic acid, bis(2-ethylhexyl) ester	0.20	C_22_H_42_O_4_	370.60
94	8343	Bis(2-ethylhexyl) phthalate	1.23	C_24_H_38_O_4_	390.60

### 4.2 Identifying the targets of AMS

**Figure 3:**
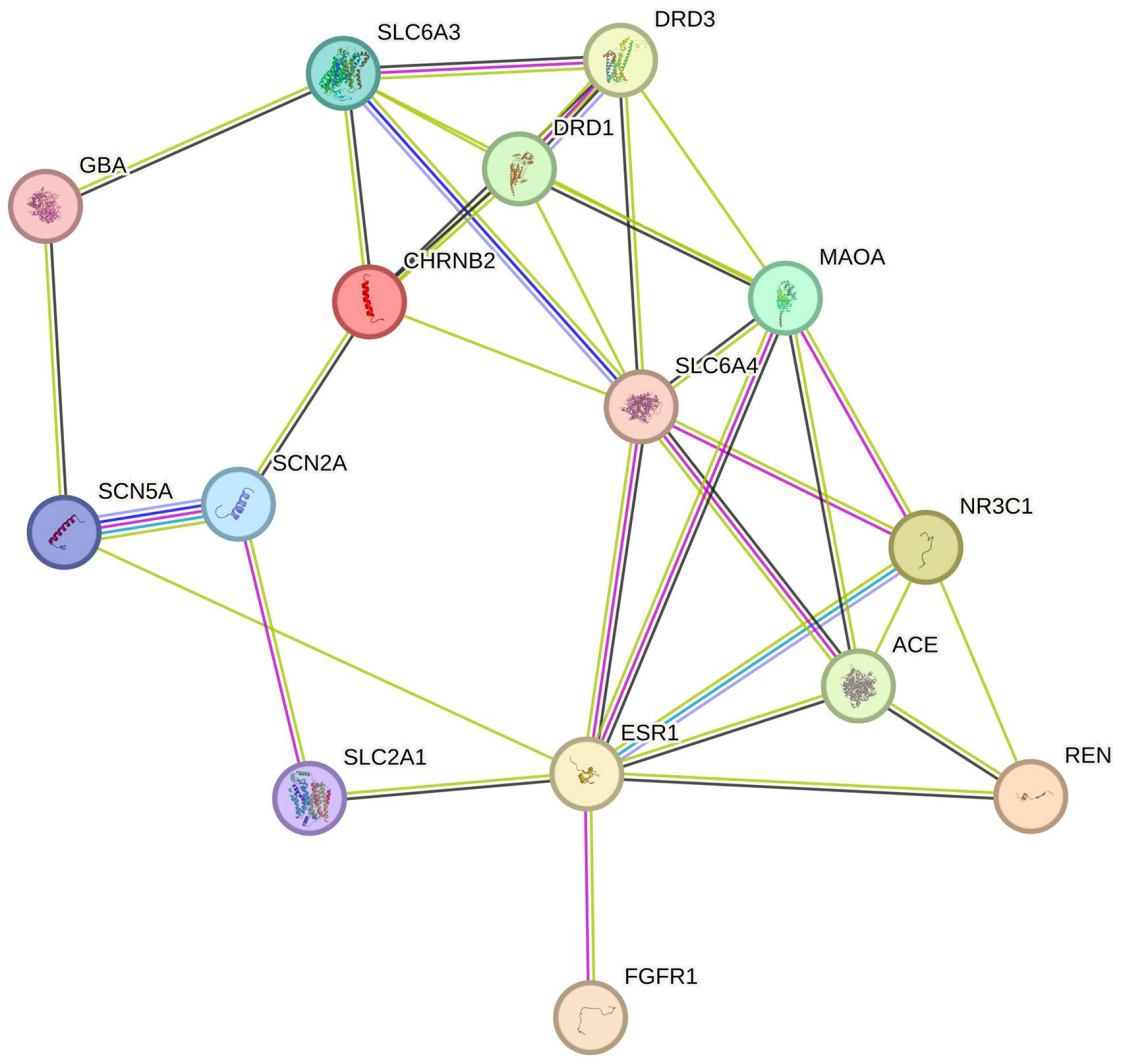
PPI network of 17 intersecting target genes.

After searching the TCMSP database based on GC–MS results identified 94 effective standardized active ingredients for sleep disorders. By integrating target data, the potential targets of the 94 chemical components of AMS were predicted, with a total of 514 target sites. Using “Sleep disorders” as the search term, 17 gene targets were obtained by compiling the sleep disorder-related disease genes identified in multiple databases according to the described screening criteria (Figure 3, Table 2).

**Table 2:** 17 Intersecting target genes.

Numberofnodes	17
Numberofedges	34
Averagenodedegreee	4
Avg.Localclustering coefficient	0.47
Expected number of edges	7
PPlenrichmentp-value	<2.88e^−14^

### 4.3 Construction and analysis of the PPI network

The 94 components of AMS and sleep disorders shared 17 nonoverlapping targets. Based on the STRING background network, a PPI network was constructed based on the potential targets of AMS in treating sleep disorders. The network contained 17 nodes and 34 edges, with an average node degree value of 4. According to the enrichment analysis, the PPI network was significantly enriched with a *p*-value <0.47. Based on the number of active components, the core targets were identified as the glucose transporter 1 (GLU1) gene SLC2A1 (31), the monooxygenase A gene MAOA (32), the synaptic-related genes SCN2A (33), and the dopamine transporter receptor SLC6A3 (34).

### 4.4 GO and KEGG analysis

**Figure 4:**
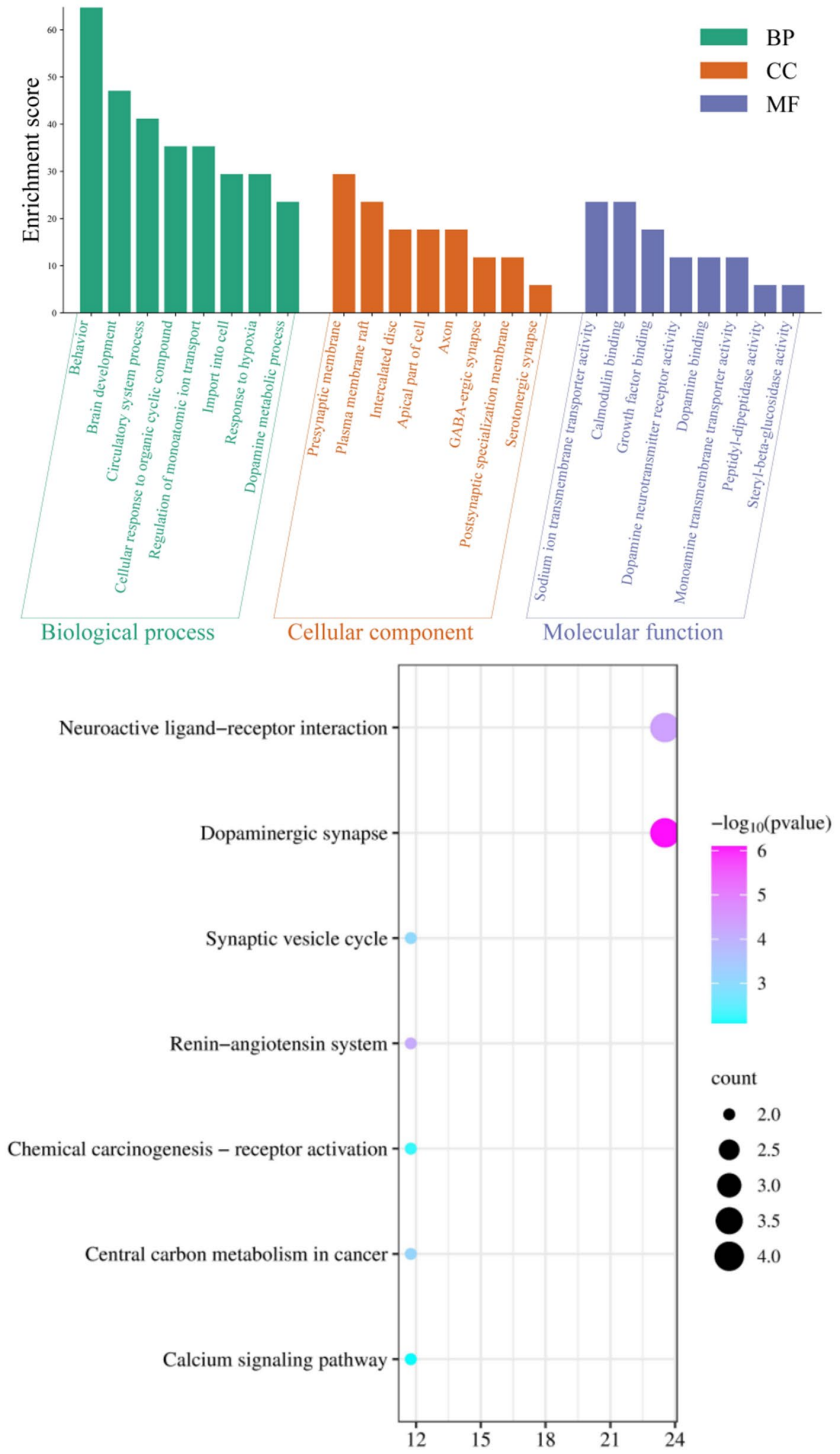
GO analysis and KEGG signal pathway bubble chart of the target points for improving sleep disorders by the AMS.

The integration of various datasets, network design, route analysis, molecular docking, scoring functions, and docking validation present computational overhead for the study, which looks into the therapeutic benefits of Agarwood Moxa Smoke (AMS) on sleep disorders. The more datasets and interactions there are, the greater the processing cost. Computational resources are needed throughout network building for data processing, visualization, and analysis. Pathway analysis entails processing substantial amounts of biological data to find essential pathways connected to the therapy of AMS. Due to the potential requirement to analyze several scoring methods, scoring functions incur additional computational complexity. Robust studies need a delicate balance between computational complexity and analytical depth. To gain a deeper understanding of the mechanism of action of AMS in the treatment of sleep disorders, in this study, we conducted GO and KEGG analyses of the 514 potential targets of AMS in the treatment of sleep disorders. The eight biological processes with the most significant enrichment by the components of AMS (sorted by *p*-value) included behavior (*p* = 2.34*10^−15^), brain development (*p* = 3.98*10^−9^), circulatory system processes (*p* = 5.01*10^−9^), cellular response to organic cyclic compounds (*p* = 2.10*10^−7^), regulation of monoatomic ion transport (*p* = 6.31*10^−7^), import into the cell (*p* = 8.32*10^−8^), response to hypoxia (*p* = 3.80*10^−7^), and dopamine metabolic processes (*p* = 1.62*10^−9^). In terms of cell components, the treatment of sleep disorders with AMS mainly involves the presynaptic membrane (*p* = 2.57*10^−8^), plasma membrane rafts (*p* = 4.37*10^−7^), intercalated disks (*p* = 2.82*10^−6^), apical parts of the cell (*p* = 2.04*10^−3^), axons (*p* = 5.01*10^−3^), GABAergic synapses (*p* = 1.00*10^−3^), postsynaptic specialization membranes (*p* = 2.19*10^−3^), and serotonergic synapses (*p* = 1.12*10^−3^). In terms of molecular function, the target genes were mainly enriched in protein cell activity and binding, including aspects such as sodium ion transmembrane transporter activity (*p* = 1.62*10^−6^), calmodulin binding (*p* = 4.27*10^−6^), growth factor binding (*p* = 5.37*10^−5^), dopamine neurotransmitter receptor activity (*p* = 2.95*10^−6^), dopamine binding (*p* = 6.17*10^−6^), monoamine transmembrane transporter activity (*p* = 1.95*10^−5^), peptidyl-dipeptidase activity (*p* = 1.12*10^−3^), and steryl-beta-glucosidase activity (*p* = 1.12*10^−3^) (Figure 4, Table 3).

**Table 3:** Gene ontology BP, CC, MF top 8 entry information.

No.	Category	Type description	*p*-value
1	Biological processes	Behavior	2.34*10^−15^
2		Brain development	3.98*10^−9^
3		Circulatory system process	5.01*10^−9^
4		Cellular response to organic cyclic compound	2.10*10^−7^
5		Regulation of monoatomic ion transport	6.31*10^−7^
6		Import into cell	8.32*10^−8^
7		Response to hypoxia	3.80*10^−7^
8		Dopamine metabolic process	1.62*10^−9^
9	Cellular components	Presynaptic membrane	2.57*10^−8^
10		Plasma membrane raft	4.37*10^−7^
11		Intercalated disk	2.82*10^−6^
12		The apical part of the cell	2.04*10^−3^
13		Axon	5.01*10^−3^
14		GABA-ergic synapse	1.00*10^−3^
15		Postsynaptic specialization membrane	2.19*10^−3^
16		Serotonergic synapse	1.12*10^−3^
17	Molecular functions	Sodium ion transmembrane transporter activity	1.62*10^−6^
18		Calmodulin binding	4.27*10^−6^
19		Growth factor binding	5.37*10^−5^
20		Dopamine neurotransmitter receptor activity	2.95*10^−6^
21		Dopamine binding	6.17*10^−6^
22		Monoamine transmembrane transporter activity	1.95*10^−5^
23		Peptidyl-dipeptidase activity	1.12*10^−3^
24		Steryl-beta-glucosidase activity	1.12*10^−3^

Enrichment through KEGG pathway analysis showed that the effect of AMS on sleep disorders was significant. The seven KEGG pathways with considerable enrichment were dopaminergic synapse (*p* = 7.94*10^−7^), the neuroactive ligand–receptor interaction (*p* = 4.47*10^−5^), the renin-angiotensin system (*p* = 7.41*10^−5^), central carbon metabolism in cancer (*p* = 7.08*10^−4^), the synaptic vesicle cycle (*p* = 8.71*10^−4^), chemical carcinogenesis-receptor activation (*p* = 6.17*10^−3^), and the calcium signaling pathway (*p* = 7.94*10^−3^), suggesting that AMS can regulate metabolic pathways related to sleep and restore the function of metabolic pathways that were impacted by insomnia (Figure 4).

**Figure 5:**
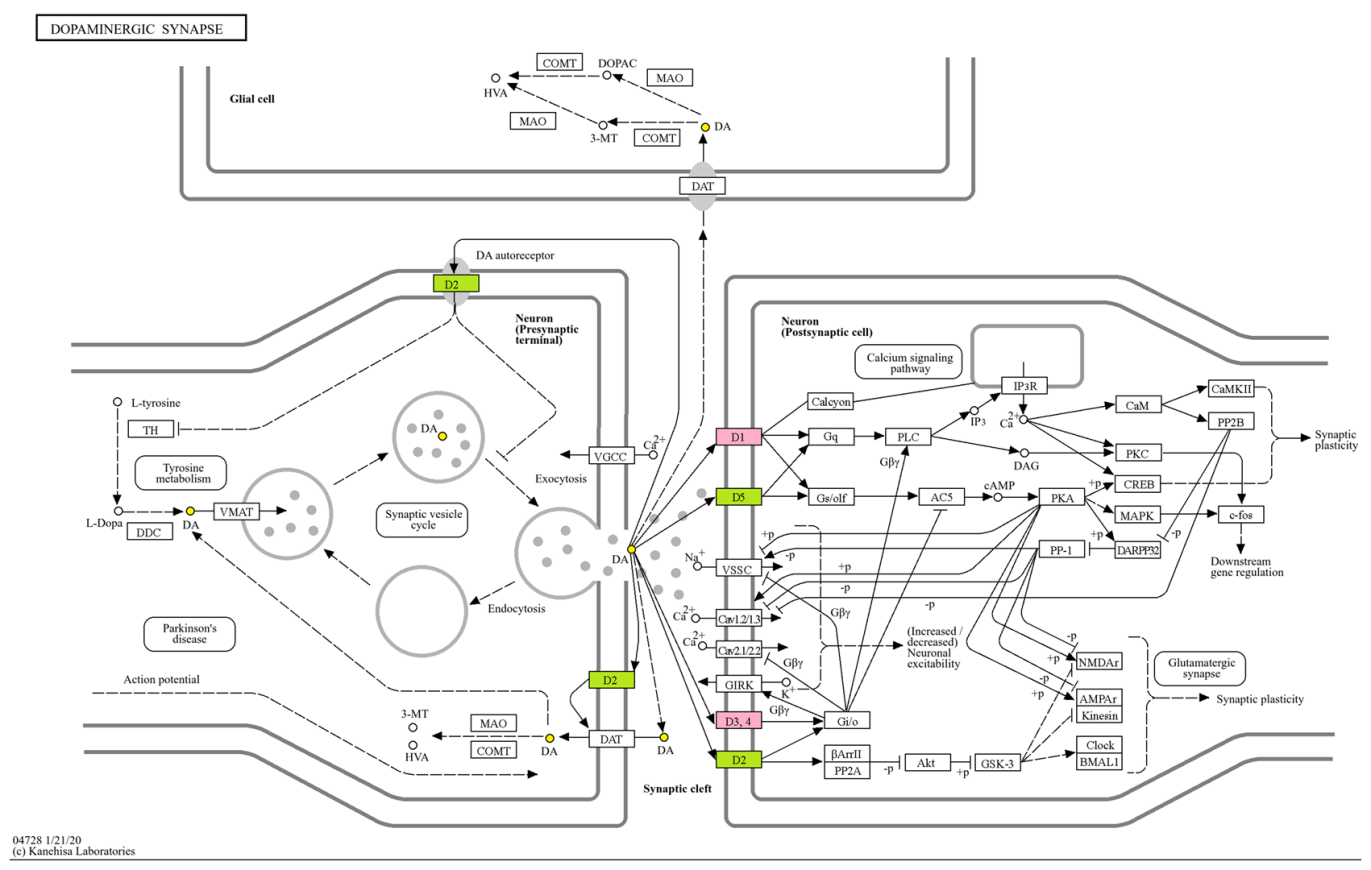
Dopaminergic synaptic pathway.

The glucose transporter gene SLC2A1, the monooxygenase A gene MAOA, the synaptic-related genes SCN2A, dopamineD1 receptor DRD1 and dopamineD3 receptor DRD3, the nicotinic acetylcholine β2 receptors CHRNB2, the 5-hydroxytryptamine transporter receptor SLC6A4, and the dopamine transporter receptor SLC6A3 are all associated with multiple pathways and promote neurotransmitter transport and neuronal excitability. The regulation of these targets by the active ingredients of AMS can simultaneously alter numerous signaling pathways related to sleep disorders, reflecting the “multi-component multi-target multi-pathway” approach. We mapped the dopamine receptor family DRD1, DRD3, and other targets for improving sleep disorders with the components of AMS to the dopaminergic metabolic signaling pathway and constructed a metabolic pathway map (Figure 5).

### 4.5 Construction of the “components-targets-action pathway” network diagram

**Figure 6:**
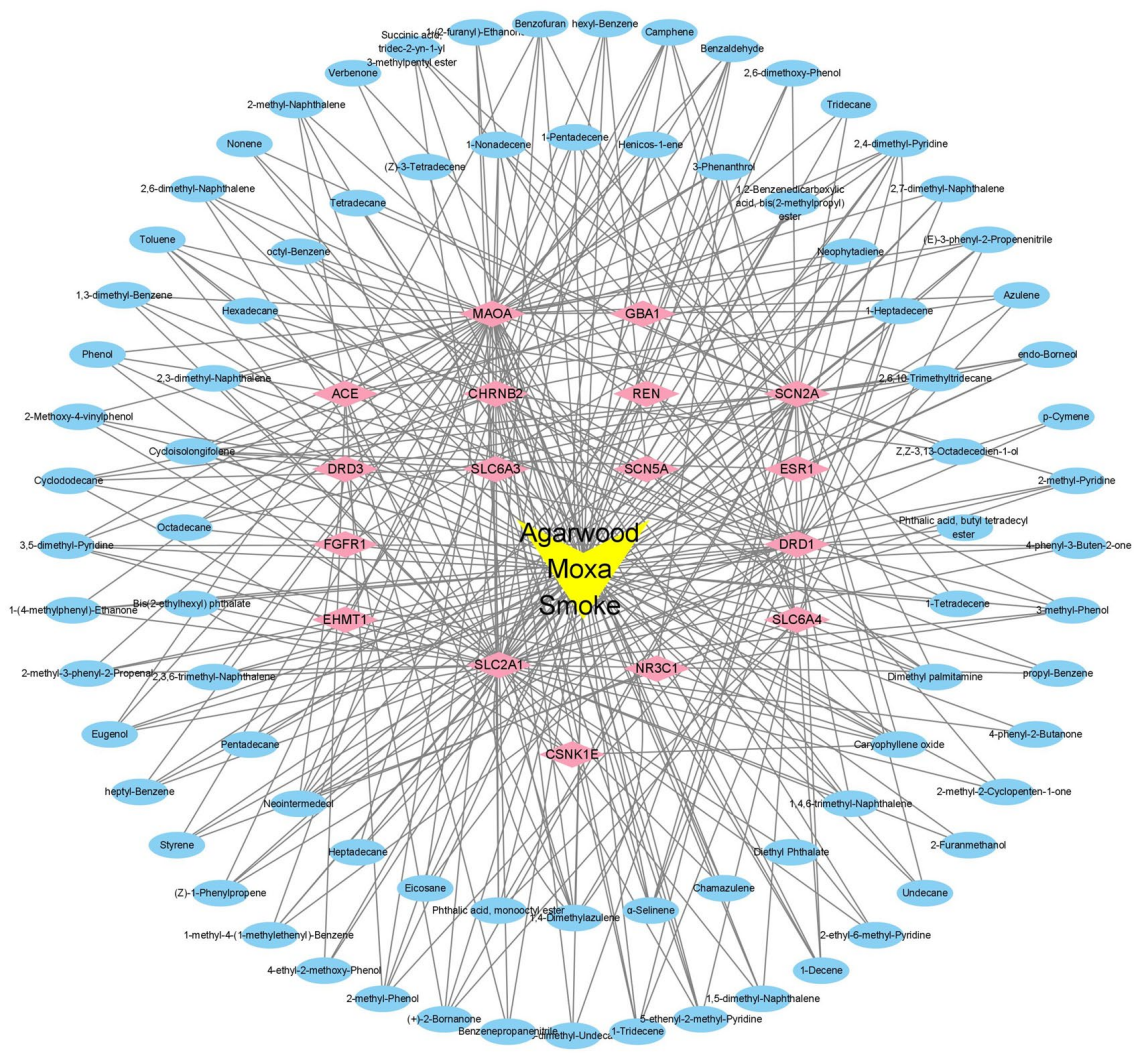
“Components-efficacy targets-action pathway network” of AMS.

With the help of Cytoscape 3.9.1 software, the active ingredients and their targets, as well as the targets involved in sleep disorders, were collected, and a network diagram of the “components-targets-action pathway” of AMS was constructed. The “component-target-pathway” network diagram of AMS intuitively displays the corresponding targets of each active ingredient in AMS. The blue circular nodes represent the 94 functional targets involved in sleep disorders, and the pink diamond nodes represent the 17 related genes that can help regulate sleep disorders. The connection between the component and the target indicates that the element can regulate the target (Figure 6).

### 4.6 Molecular docking results

**Figure 7:**
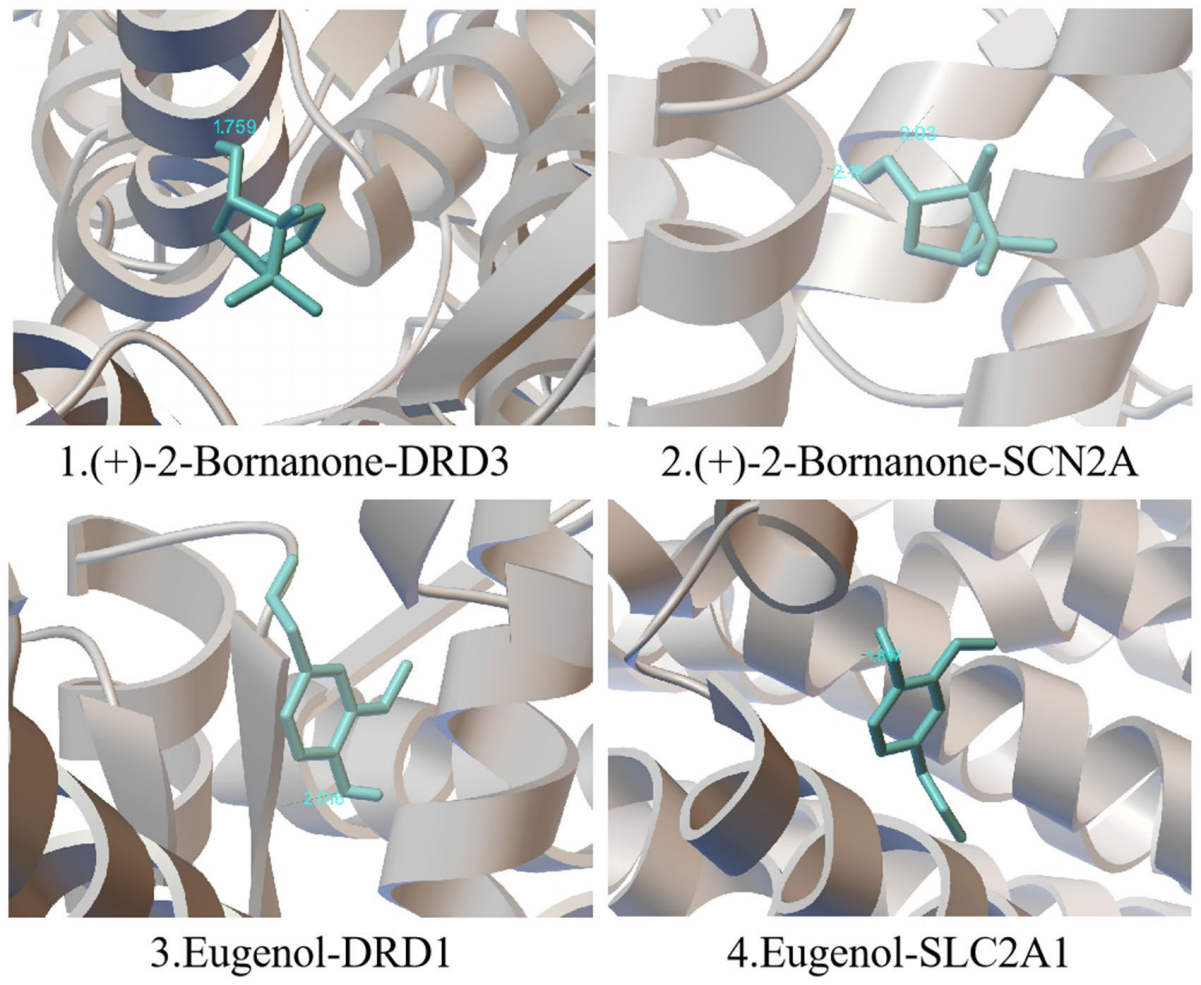
Schematic diagram of simulated docking between the main active ingredients and core targets of AMS.

The 5 main components (p-cymene, (+)-2-bornanone, endo borneol, caryophyllene oxide, and eugenol) of AMS related to the 5 key targets (SLC2A1, MAOA, SCN2A, DRD1, and DRD3) in the “component-target-pathway” network. The protein crystal structure of the target was obtained from the PDB database (PDB ID numbers: 6THA, 6EZZ, 4RLY, 7JVP, and 7CMV). The molecular docking results showed that the binding energies of (+)-2-bornanone with DRD3 and SCN2A and eugenol with DRD1 and SLC2A1 were less than-5 kJ·mol^−1^, indicating that the identified components have good binding with the targets and the main active components of AMS can improve sleep disorders via multiple targets. Some receptor-ligand binding patterns are shown in Figure 7 and Table 4.

**Table 4:** Main active ingredients and core targets of AMS.

Number	Chemical compounds	Core targets	Binding energy (kcal/mol)
1	(+)2–Bornanone	DRD3	−4.55
2	(+)2–Bornanone	SCN2A	−3.97
3	Eugenol	DRD1	−4.76
4	Eugenol	SLC2A1	−4.82

According to the study, qing moxa smoke contains 294 chemicals that may be hazardous to the kidney, liver, and heart. It highlights the complexity of the components of moxa smoke. It recommends that to protect the health of patients and practitioners, moxibustion rooms should have artificial or mechanical ventilation (35). The samp8 mice were divided into six groups, and the anti-aging effects of moxa smoking were investigated. In comparison to the normal control group, the results demonstrated a considerable drop in cerebral 5-ht, da, and ne levels. On the other hand, 5-ht and ne levels were greater in groups l2, m1, and m2, whereas da levels were higher in l2 and m2 (36). However, Table 5 shows the comparison between existing and proposed techniques as compared with the AMS results.

**Table 5:** Comparison between existing and proposed study.

Reference number	Methods used	Results attained
(35)	Qing moxa smoke	89%
(36)	SAMP8 mice	91.2%
Proposed method	AMS	92.3%

## 5 Discussion

The GC–MS analysis results show that there are 9 main active ingredients in AMS, including p-cymene (1.09%), eucalyptol (0.81%), (+)-2-bornanone (0.85%), endo-borneol (1.24%), α-terpineol (1.11%), eugenol (0.46%), caryophyllene (2.28%), caryophyllene oxide (1.79%) and n-hexadecanoic acid (0.40%). These ingredients generally have antioxidant, anti-inflammatory, antibacterial, and antitumor effects. In addition, p-cymene has analgesic and neuroprotective effects (37); eucalyptol can reduce neural excitability and has soothing, neuroprotective, anti-anxiety, and antidepressant effects (38). The main chemical components of AMS have activities such as pain relief and sedation, which can be used to treat sleep disorders. However, many other components of AMS and their capabilities have not been investigated or verified, which will require a more thorough study.

This study showed that SLC2A1, SLC6A3, SLC6A4, MAOA, SCN2A, DRD1, DRD3, and CHRNB2 are the core targets for AMS treatment of sleep disorders. The dopamine receptor family, including DRD1, DRD3, and SLC6A3, mainly acts on dopamine (DA), while MAOA and SLC6A4 primarily affect serotonin (5-HT). SCN2A is a subunit-encoding voltage-gated sodium ion channel in the central nervous system that is widely distributed at the beginning of the axons of glutamate neurons and is involved in the regulation of hippocampal replay within sharp wave ripples (SPW-Rs), which are essential for memory (39). SCN2A can control excitatory synaptic input (40), thus regulating neuronal excitability. DRD1 is a receptor for the excitatory neurotransmitter DA, and its activity is mediated by the G protein that activates adenylate cyclase. DRD1 can increase DA levels through the DRD1 MeCP2 BDNF TrkB signaling pathway, leading to insomnia (41). Sleep-related epilepsy can be caused by mutations in genes such as CHRNB2, which encodes the nAChR subunit and is widely expressed in the forebrain (42). SLC6A4 is a 5-HT transporter that can help maintain 5-HT homeostasis in the central nervous system and affect sleep by regulating 5-HT transport. SLC6A4 transports 5-HT from the extracellular compartment to the cytoplasm through the exchange of Na^+^ during the electroneutral transport cycle, thereby limiting the intercellular signal transduction of 5-HT (43). In the raphe neurons of the brainstem, the uptake of 5-HT from the synaptic gap to the presynaptic end is regulated, thereby terminating the transmission of 5-hydroxytryptamine signals at the synapse. In addition, mutations in the SLC6A4 and MAOA genes can induce structural and functional abnormalities in the dorsal raphe nucleus (DRN) and amygdala, thereby interfering with rapid eye movement (REM) sleep (44). In treating sleep disorders, AMS can mediate the expression of the genes above, thereby affecting emotions and sleep by controlling the transport and metabolism of related proteins and neurotransmitters, the permeability and exchange of Na^+^, and the transmission of synaptic signals.

The results of KEGG analysis showed that the mechanism of action of AMS in treating sleep disorders mainly involves the dopamine synaptic pathway, the neuroactive ligand–receptor interaction, the renin-angiotensin system, the synaptic vesicle cycle pathway, and other signaling pathways. Among them, dopaminergic synapses are chemical synapses that play a crucial role in emotional disorders and can affect the connections of all members of the axonal protein superfamily of transmembrane molecules that play essential roles in neuropsychiatric disorders and excitatory cells. Excessive activation of the renin-angiotensin system pathway can lead to disturbances in the internal environment, increased reabsorption of Na^+^ by the renal tubules, and elevated levels of renin and angiotensin, leading to elevated blood pressure, insomnia, anxiety, depression, and inflammation (45, 46). When insomnia occurs in the human body, the transmission of excitation signaling pathways is enhanced, and the content of molecules related to this pathway also increases. GO analysis of the active components of AMS showed that the core target genes were involved in processes such as behavior, brain development, circulatory system processes, and cellular responses to organic cyclic compounds. Therefore, based on the above results, the active ingredients of AMS can improve the behavior of individuals with sleep disorders and help maintain normal brain development and function by regulating the circulatory system; meanwhile, the active ingredients of AMS can communicate and activate various pathways through signal transduction and activating transcription factors, thereby controlling the levels of related neurotransmitters, hormones, signal molecules, and other substances.

### 5.1 Limitations and future scope

Using molecular docking and network pharmacology, the study explores the potential use of AMS in treating sleep disorders. However, *in vitro* and *in vivo* studies still need to be improved for better results. To comprehend the molecular processes behind the therapeutic benefits of AMS, future research should concentrate on thorough experimental investigations, clinical trials, and the integration of multi-omics data. Enhancing comprehension of AMS’s therapeutic potential and creating novel treatment approaches may be accomplished by evaluating patient-centered outcomes and quality-of-life metrics.

## 6 Conclusion

The average amount of time people spend sleeping is steadily declining, the number of people who have insomnia is rising, and the causes of sleep disorders are becoming more complicated. Traditional Chinese medicine’s diagnosis and treatment philosophy, based on syndrome differentiation and numerous potent ingredients, offers effective treatments for various insomnia-related conditions. Clinical experience has demonstrated that AMS is beneficial for qi circulation, pain relief, regulating meridians and kidneys, and calmness. In this study, we used GC–MS technology to identify the chemical components in AMS. The obtained compounds were subjected to network pharmacology analysis, and 94 active components of AMS were identified. Five hundred fourteen disease targets, 17 shared active component regulation targets, and sleep disorder-related targets were identified. A “components-targets-pathways” network for AMS and GO was established. KEGG analyses were utilized to speculate that AMS may regulate sleep disorders through the following 7 pathways: dopaminergic synapse, the neuroactive ligand–receptor interaction, the renin-angiotensin system, central carbon metabolism in cancer, the synaptic vesicle cycle, chemical carcinogenesis-receptor activation, and calcium signaling pathways. Based on the specific roles of targets and components, 8 key targets were selected, including 9 potential active monomers. Molecular docking was carried out, and DRD3 and SCN2A showed good binding with (+)-2-Bornanone, and DRD1 and SLC2A1 showed good binding with eugenol. In this study, we explored the potential mechanisms underlying the calming and tranquilizing effects of AMS in sleep disorders from the following two aspects: material component analysis and network pharmacology, thereby providing a theoretical basis for further exploration and subsequent experimental research to evaluate the clinical application of AMS to improve sleep.

## Data availability statement

The original contributions presented in the study are included in the article/supplementary material, further inquiries can be directed to the corresponding authors.

## Author contributions

NC: Writing – original draft, Data curation. YX: Writing – original draft, Formal analysis. WW: Writing – original draft, Funding acquisition. SC: Writing – original draft, Investigation. MZ: Writing – original draft, Methodology. YS: Writing – original draft, Project administration. YL: Writing – original draft, Conceptualization.

## Footnotes

^1^ https://old.tcmsp-e.com/tcmsp.php

^2^ https://pubchem.ncbi.nlm.nih.gov/

^3^ http://www.swisstargetprediction.ch/

^4^ https://www.ebi.ac.uk/chembl/

^5^ http://www.uniprot.org

^6^ https://www.genecards.org/

^7^ https://www.disgenet.org/

^8^ https://david.ncifcrf.gov/

^9^ http://jvenn.toulouse.inra.fr/app/example.html

^10^ https://cn.string-db.org/

^11^ https://Metascape.org/

^12^ https://www.rcsb.org/
